# Iodine in Health and Disease: A Comprehensive Review

**DOI:** 10.3390/nu18081262

**Published:** 2026-04-16

**Authors:** Tea Delić, Sandra Karanović Štambuk

**Affiliations:** 1 General Hospital Zadar, 23000 Zadar, Croatia; 2 School of Medicine, University of Zagreb, 10000 Zagreb, Croatia; 3 Department of Nephrology, Arterial Hypertension, Dialysis and Transplantation, University Hospital Center Zagreb, 10000 Zagreb, Croatia

**Keywords:** iodine, iodine deficiency disorders, iodine excess, salt iodization, thyroid hormone synthesis

## Abstract

Iodine is an essential micronutrient required for the synthesis of thyroid hormones and the maintenance of metabolic, neurodevelopmental and immune function. As iodine cannot be synthesized endogenously, adequate intake depends on dietary sources and environmental availability. Despite decades of progress in improving iodine supply, both iodine deficiency and excess remain significant global public health challenges. This review summarizes iodine physiology, covering both its role in thyroid hormone synthesis and emerging evidence for extrathyroidal immunomodulatory and antioxidant actions. It summarizes major dietary sources, global intake patterns and current approaches to iodine status assessment, including urinary biomarkers, salivary iodide measurement and dietary screening tools. The clinical consequences of iodine imbalance are examined, ranging from goiter, hypothyroidism and impaired neurocognitive development associated with deficiency, to iodine-induced thyroid dysfunction, autoimmunity and adverse systemic effects linked to excess intake. Special attention is given to vulnerable populations, particularly pregnant women and infants. This review further evaluates public health strategies, including salt iodization and targeted supplementation, while addressing the emerging challenge posed by salt-reduction initiatives. Achieving optimal iodine intake remains essential for thyroid health and population well-being, underscoring the need for coordinated monitoring and policy adaptation.

## 1. Introduction

Globally, despite the remarkable progress that has been made in improving iodine intake and reducing iodine inadequacy, iodine deficiency remains a public health concern in certain populations, as recognized by the World Health Organization (WHO) [1]. Iodine is an essential micronutrient that serves as a structural component of thyroid hormones and is required for normal physiological function. Since it cannot be synthesized by the human body, it must be obtained exogenously through the diet. Unlike most essential nutrients, iodine status is influenced less by socioeconomic development and more by geographical factors, particularly the iodine content of local food supplies, which depends on the concentration of iodine in soil and water. Consequently, low environmental iodine levels result in iodine-deficient plants and animals [1].

Through its crucial role in the synthesis of thyroid hormones–triiodothyronine (T3) and thyroxine (T4), iodine regulates numerous physiological processes, including metabolism, growth and neurological development. Therefore, both iodine deficiency and excess are associated with significant health issues [2].

Globally, iodine deficiency remains a public health concern, despite substantial progress in iodine nutrition and widespread implementation of iodized salt programs [3]. Iodine deficiency can result in disorders such as goiter, hypothyroidism, and cognitive impairment. Conversely, excessive iodine intake, often from high-iodine diets or through the introduction of iodine-supplying interventions when they are unneeded, can disrupt thyroid function, leading to conditions such as hyperthyroidism and autoimmune thyroiditis, especially in individuals with pre-existing thyroid disorders [4].

This comprehensive review aims to elucidate the physiological role of iodine, its dietary sources, the global prevalence of its deficiency and excess, and the associated health implications. Additionally, it explores public health strategies for attaining appropriate iodine status and discusses emerging research on iodine’s role in chronic diseases.

## 2. Iodine’s Physiological Role and Mechanisms of Action

### 2.1. The Role of Iodine in Thyroid Hormone Synthesis

Iodine’s primary physiological role is to enable thyroid-hormone (TH) synthesis. After oral intake, iodine is reduced to iodide (I^−^) and absorbed in the duodenum [5]. I^−^ is actively taken up by the sodium-iodide symporter (NIS) on the basolateral membrane of thyroid follicular cells, which co-transports two Na^+^ ions per I^−^ using the Na^+^/K^+^-ATPase gradient [6,7]. NIS is also expressed in several extrathyroidal tissues, including lactating breast, placenta and salivary glands [8,9]. Once inside the thyrocyte, I^−^ reaches the follicular lumen via apical transporters such as pendrin, calcium-activated chloride channel (CaCC) and cystic fibrosis transmembrane conductance regulator [10,11]. There, thyroid peroxidase (TPO) oxidizes I^−^ to iodine (I_2_) with H_2_O_2_ from dual oxidase 2 and organifies it onto tyrosine residues of thyroglobulin (Tg), producing monoiodotyrosine (MIT) and diiodotyrosine (DIT) [10,11]. Coupling of MIT and DIT yields T3, whereas coupling of two DIT molecules yields T4 [11].

TH release occurs following endocytosis of iodinated Tg, lysosomal proteolysis and transport of T3 and T4 into the bloodstream via monocarboxylate transporter 8 [11]. Thyroid-stimulating hormone (TSH) stimulates NIS expression and the entire synthetic cascade through cyclic adenosine monophosphate signaling [5]. Competitive inhibitors such as perchlorate and thiocyanate block NIS and thereby reduce iodine uptake [5,6].

Excess iodine transiently suppresses thyroid hormone synthesis (Wolff–Chaikoff effect); normal thyroid function resumes after the so-called “escape”, which reflects the restoration of hormone synthesis following adaptation to excess iodine through reduced intrathyroidal iodine levels and decreased NIS activity [12]. This suppression is mediated by inhibition of TPO and down-regulation of NIS expression [13]. In contrast, iodine deficiency lowers the DIT-to-MIT and T4-to-T3 ratios, thereby impairing TH production [7,10].

### 2.2. Immunomodulatory and Antioxidant Roles of Iodine

Iodine is not only essential for thyroid hormone synthesis, but also exerts direct immunomodulatory and antioxidant effects on many tissues. Human leukocytes express iodide transporters (NIS, pendrin) and thyroid-related proteins. Exposure to sodium iodide markedly alters their transcriptome, enhancing chemokine expression and both pro- and anti-inflammatory cytokine production [14,15].

Molecular iodine (I_2_) further modulates immunity in a context-dependent manner: in non-antigenic damage it suppresses neutrophil and mast-cell degranulation and reduces macrophage nitric oxide and tumor necrosis factor-alpha release, whereas in the presence of extracellular pathogens, I_2_ enhances neutrophil phagocytosis and chemotaxis and promotes T helper 2 (Th2) cytokine production, including interleukin (IL)-6, IL-8, and IL-10, supporting regulatory T cell (Treg) differentiation [15,16]. Conversely, during intracellular antigen exposure, such as viral infection or malignancy, I_2_ promotes a T helper 1 (Th1)-dominant response characterized by increased IL-2 and interferon-gamma production and activation of natural killer cells, cytotoxic T lymphocytes, and classically activated M1 macrophages [14,15,16].

I_2_ also acts as a potent antioxidant. It directly scavenges reactive oxygen species, induces type II antioxidant enzymes and can inactivate pro-inflammatory pathways [16]. In the salivary lactoperoxidase system, acute iodine loads raise iodide and hypoiodous acid (HOI) concentrations; through peroxidase-mediated reactions, iodine contributes to antimicrobial defense by generating HOI, a reactive iodine species with antimicrobial and antioxidant properties that may protect airway surfaces against viruses and bacteria [17].

Overall, iodine exerts significant extrathyroidal effects, particularly in immune regulation and antioxidant defense. Its immunomodulatory actions include balancing Th1 and Th2 responses, promoting Treg differentiation, and modulating inflammasome activity, including upregulation of NLRP3—the nucleotide-binding oligomerization domain-like receptor family pyrin domain containing 3 - inflammasome in peripheral blood mononuclear cells [18]. In addition, iodine contributes to antioxidant defense by neutralizing reactive oxygen species, enhancing antioxidant enzyme expression [16]. These mechanisms suggest that adequate iodine intake could contribute to both immune competence and oxidative stress resistance.

## 3. Iodine Sources and Dietary Intake

Iodine is naturally present in a variety of foods, although its concentration is highly variable depending on soil content, agricultural practices and food processing methods [1,19]. Due to geological factors such as glaciation, flooding and soil erosion, the iodine content of soil varies greatly between regions. In the seas and oceans, iodide is converted into elemental iodine, which enters the atmosphere and, as part of an ecological cycle, is returned to the land via precipitation in the form of rain and snow. However, this cycle is slow and inconsistent and the redistribution of iodine through atmospheric deposition is insufficient to adequately replenish iodine poor soils and freshwater [1].

Major dietary sources include seafood (especially marine fish and seaweed), dairy products and eggs [20]. The iodine content in grains and vegetables varies widely depending on the iodine concentration in the soil where they are grown [1].

When dietary iodine intake from food and drinking water is insufficient, the fortification of salt with iodine has been an effective public health intervention for more than a century and is currently implemented in over 120 countries worldwide [3,21]. Even though salt iodization represents the most effective public health measure to prevent iodine deficiency in populations with insufficient dietary iodine intake, one must keep in mind that the need for iodized salt depends on the baseline iodine supply from dietary and environmental sources, and in iodine-sufficient or iodine-rich settings, iodine intake should be carefully monitored to avoid excessive exposure [4]. Table salt is fortified with potassium iodate, with the WHO recommending 20–40 mg iodine per kg of salt to ensure adequate daily intake [22]. These iodine concentrations were selected to provide sufficient iodine intake in populations in which iodized salt is the main source of iodine, assuming an average daily salt consumption of approximately 5–10 g [22]. Some countries have also implemented fortification in other food products, such as bread or condiments, or use iodized oil capsules or injections in at-risk populations [23]. The recommended daily iodine intake varies depending on age and physiological status. These recommendations, issued by the WHO, are summarized in Table 1 [22]. These values represent recommended nutrient intakes (RNIs) for individuals and do not correspond to population-level requirements, which are typically defined using estimated average requirements and are generally lower than RNI values (Table 2) [22,24].

## 4. Methods for Assessing Iodine Intake and Status

Methods used to assess iodine nutrition primarily reflect iodine intake rather than its physiological effects. As urinary iodine concentration reflects recent iodine intake rather than functional iodine status, complementary biomarkers are required to evaluate the impact of iodine on the hypothalamic–pituitary–thyroid axis [7]. In this context, thyroid function parameters such as TSH, free thyroxine (fT4), and Tg can provide insight into the physiological effects of iodine. However, these biomarkers are influenced by multiple factors beyond iodine intake, including age, pregnancy, inflammation, and underlying thyroid disorders, which limits their specificity for assessing iodine status, particularly in individuals and populations with mild iodine deficiency [7,22].

### 4.1. Dietary Salt Analysis

Iodine in commercial and bulk salts can be measured by iodometric titration, which yields quantitative values, or by a spot-testing kit, which is used to qualitatively determine whether salt is iodized [25].

### 4.2. Urinary Biomarkers

The most widely used biomarker in iodine surveillance is spot urinary iodine concentration (UIC), which is measured spectrophotometrically (Sandell–Kolthoff reaction) or by titration. Since more than 90% of ingested iodine is excreted in urine, UIC reflects recent iodine intake and is particularly useful for assessing iodine nutrition at the population level [25,26]. However, because UIC reflects only recent iodine intake and is subject to substantial day-to-day intra-individual variation, spot UIC is not suitable for diagnosing iodine status in individuals. For individual-level evaluation, repeated measurements, 24 h urinary iodine excretion, dietary assessment, and thyroid function markers may provide a more informative overall assessment, depending on the clinical context [25,26,27]. Since urinary biomarkers primarily reflect iodine intake rather than its physiological effects, complementary biomarkers are required to assess the functional impact of iodine on the hypothalamic–pituitary–thyroid axis [7]. At the population level, median UIC values are used to classify iodine intake, with <100 µg/L indicating insufficient intake, 100–199 µg/L adequate intake, and ≥300 µg/L excessive intake in adults [25].

### 4.3. Salivary Iodide Concentration (SIC)

Saliva sampling offers a low-burden alternative to urine collection. Salivary iodide concentration correlates with urinary iodine concentration (UIC) and urinary iodine excretion (UIE) (r = 0.19–0.90), as well as with dietary iodine intake, particularly when samples are collected after the evening meal. Proposed population cut-off values include <93 µg/L for deficiency, 93–225 µg/L for adequacy and >225 µg/L for excess [28].

### 4.4. Digital Dietary Screener

An iodine-specific digital screener (I-screener) estimates dietary intake and classifies individuals into deficient, adequate, or excessive intake categories. It demonstrates acceptable but limited agreement with 24 h dietary recall (Cohen’s κ = 0.187), indicating a persistent risk of misclassification [27].

### 4.5. Combined Approaches

Best practice involves integrating dietary assessment tools (food-frequency questionnaires or 24 h recalls) with urinary measurements and, when feasible, salt iodine analysis, to triangulate iodine intake [25]. In addition, the inclusion of thyroid function biomarkers such as TSH, fT4 and Tg may provide complementary information on the physiological effects of iodine, although their interpretation remains context-dependent [7].

## 5. Iodine Status and Thyroid Health

### 5.1. Iodine Deficiency and Thyroid Dysfunction

Iodine deficiency (ID) remains the leading preventable cause of intellectual disability and thyroid disease worldwide. It develops when dietary iodine intake is too low to sustain normal synthesis of T3 and T4 [29]. The resulting iodine deficiency disorders (IDDs) encompass goiter, hypothyroidism, and a continuum of neurodevelopmental impairments that originate in utero and may persist throughout childhood [30].

#### 5.1.1. Goiter

When iodine intake and status are chronically inadequate, the thyroid cannot produce sufficient amounts of T4, leading to a decline in circulating T4 levels and a compensatory increase in TSH secretion. Persistently elevated TSH drives iodine trapping, thyroid peroxidase activity and, importantly, the proliferation (hyperplasia) and enlargement (hypertrophy) of thyroid follicular cells. This chronic stimulation produces a diffuse, often symmetric enlargement of the gland—clinically recognized as a goiter [7].

If iodine deficiency persists, the gland’s adaptive growth can become irregular, giving rise to a nodular or multinodular goiter, especially in adults living in iodine-deficient regions [31]. The goiter itself may be mild (reversible by re-iodization) or may progress to larger nodular forms that can later develop autonomous hormone production (toxic nodular goiter) or cause a predisposition to hyper- or hypothyroidism [32].

Thus, the pathophysiology of goiter in iodine deficiency reflects a compensatory feedback loop in which reduced iodine availability lowers T4 production, resulting in increased TSH secretion, thyrocyte hyperplasia and hypertrophy, and thyroid enlargement (goiter). With prolonged deficiency, this adaptive response may progress to nodular thyroid disease.

#### 5.1.2. Hypothyroidism

Hypothyroidism produces a broad spectrum of signs and symptoms, since thyroid hormone deficiency slows metabolism in virtually every organ system. Generalized fatigue, lethargy and reduced exercise tolerance are among the most common complaints [33,34]. Weight gain despite unchanged diet, often with a characteristic “puffy” appearance, results from decreased basal metabolic rate [33]. Cold intolerance and feeling “chilled” in normal environment reflects reduced thermogenesis [33,34]. Dermatologic changes include dry, coarse skin, hair loss, brittle nails and a hoarse voice due to mucopolysaccharide deposition in the vocal cords [34,35]. Gastrointestinal slowing leads to constipation and, in severe cases, gastroparesis [35]. Neurocognitive effects range from mild concentration difficulties and memory lapses to severe mental slowing; untreated disease can cause myxedema coma, a life-threatening state with profound hypothermia, bradycardia and altered consciousness [36]. Cardiovascular manifestations comprise bradycardia, reduced cardiac output, diastolic hypertension and dyslipidemia, increasing long-term atherosclerotic risk [33,37]. Reproductive disturbances such as menstrual irregularities, infertility and reduced libido are also frequent [33].

Since many of these manifestations are nonspecific, clinicians rely on a combination of symptom assessment and laboratory confirmation (elevated TSH with low free T4) to diagnose and manage hypothyroidism effectively [35].

#### 5.1.3. Neurocognitive Effects

Hypothyroidism reduces the availability of thyroid hormones essential for neuronal metabolism, synaptic plasticity, and myelination, resulting in a broad pattern of neurocognitive impairment. Affected individuals commonly exhibit specific memory deficits, particularly in verbal and visual recall, even after short-term withdrawal of levothyroxine during hormone replacement therapy [38]. Attention, processing speed, and executive function are also compromised, contributing to the “brain-fog” sensation frequently reported by patients [39]. Mood disturbances including depression and anxiety often co-occur and may further exacerbate cognitive dysfunction [40].

In older adults, overt hypothyroidism is linked to measurable decline in global cognition and may increase the risk of dementia; several longitudinal studies have reported that low serum TSH predicts faster cognitive decline in the very old [41]. Subclinical hypothyroidism, despite milder hormone changes, is associated with subtle deficits in memory and processing speed and may elevate the long-term risk of cognitive deterioration [42,43].

Mechanistically, reduced thyroid hormone levels lower brain-derived neurotrophic factor expression, impairing hippocampal neurogenesis and synaptic strength, which underlies many of the observed memory and learning problems [44]. Collectively, these findings underscore the importance of early detection and optimal thyroid hormone replacement to preserve neurocognitive health.

In school-age children, moderate iodine deficiency is associated with a mean reduction of approximately 15 points in full-scale IQ compared with adequate iodine intake [45]. Meta-analyses of heterogeneous study designs similarly estimate IQ losses of 6.9–10.2 points in iodine-deficient children [46]. Maternal ID also adversely affects offspring neurodevelopment, increasing the risk of fine-motor, problem-solving, and communication delays at 1 and 3 years of age [47].

### 5.2. Iodine Excess and Thyroid Dysregulation

Common sources of excess iodine include iodine-rich diets, iodized salts, iodine-containing supplements, amiodarone, and iodinated contrast media used in medical imaging.

Excess iodine can affect thyroid function through several mechanisms. High iodine exposure may trigger the Wolff–Chaikoff autoregulatory response, transiently reducing iodine uptake and thyroid hormone secretion. Failure to escape this effect can result in persistent hypothyroidism, particularly in susceptible individuals [48,49]. Conversely, abrupt iodine overload in iodine-deficient individuals may precipitate the Jod–Basedow phenomenon, leading to hyperthyroidism [50]. Chronic over-iodization, defined as urinary iodine concentrations exceeding 300 µg/L at the population level, has been associated with increased odds of overt and subclinical hypothyroidism, as well as a higher prevalence of thyroid autoantibodies, and should not be interpreted as a marker of iodine status at the individual level [3,51]. Excess iodine exposure has been associated with autoimmune thyroid disease and thyroid dysfunction. Systemic absorption of iodine from povidone-iodine antiseptics, particularly when applied to large wounds, mucosal surfaces, or in vulnerable populations, may contribute to renal toxicity and metabolic disturbances. Evidence also suggests possible associations with cardiovascular risk, neurotoxicity at extreme exposures, and promotion of papillary thyroid carcinoma through BRAF-mediated pathways [52].

Certain populations are particularly vulnerable to iodine excess, including children, pregnant or lactating women, older adults and individuals with pre-existing thyroid autonomy. The tolerable upper intake level for iodine in adults is set at 1100 µg/day according to the Institute of Medicine. International authorities such as the Joint FAO/WHO Expert Committee on Food Additives propose a general upper limit of 1 mg/day, while European agencies recommend lower thresholds ranging from 500 to 600 µg/day [51,53,54,55].

Although many individuals tolerate very high intakes without overt clinical effects, a clear dose–response exists for thyroid dysfunction and some systemic outcomes. This underscores the importance of careful monitoring of iodine status, particularly in the context of iodinated contrast exposure or high-dose supplementation [4,51,54].

## 6. Iodine Requirements Across Population Subgroups

The populations with the highest risk of ID are those with the greatest physiological requirements and lowest dietary iodine intake, including:

1. Pregnant and lactating women who need 2–3 times the usual iodine intake to supply the fetus and breast milk; inadequate intake raises the risk of stillbirth, miscarriage, cretinism and reduced offspring IQ [56].

2. Infants and young children who depend on maternal iodine (via placenta or breast milk) and have limited dietary diversity; deficiency can cause severe neurodevelopmental delay and hypothyroidism [57].

3. Residents of iodine-deficient geographic zones such as mountainous or inland areas of Africa, South-East Asia, the Himalayas, the Andes and parts of the WHO European Region where dairy consumption is falling face chronic low intake [58].

4. People adhering to plant-based diets (vegan or vegetarian) [59].

## 7. Iodine in Pregnancy and Fetal Development

### 7.1. Physiological Demand

During pregnancy, iodine requirements increase by approximately 65% (to about 250 µg/day) to meet the demands of increased maternal thyroid hormone production, enhanced renal iodine clearance, and transplacental transfer of iodine to the fetus [22]. This increased requirement is supported by physiological evidence demonstrating enhanced iodine turnover and increased thyroidal iodine uptake during pregnancy [56]. These changes reflect key adaptations of iodine metabolism. During lactation, iodine requirements remain elevated due to active transport of iodide into breast milk via the sodium–iodide symporter, ensuring adequate iodine supply for the infant [8,22].

### 7.2. Consequences of Iodine Deficiency and Insufficient Intake

At the population level, insufficient iodine intake during pregnancy has been associated with poorer neurodevelopmental outcomes in offspring, including lower mental development index scores in infancy and increased risk of language or fine-motor delays [57,60]. In a systematic review of 12 studies, four identified a negative association between low urinary iodine concentrations and cognitive performance in children, while the overall body of evidence was consistent with detrimental effects of iodine deficiency [57,61].

### 7.3. Excess Consequences

Excessive iodine exposure during pregnancy has also been associated with adverse neurodevelopmental outcomes, including a higher risk of developmental delay and language delay in some studies [62]. A U-shaped association between maternal iodine status and offspring neurodevelopment has been repeatedly described [56,63].

### 7.4. Population Status

Several cohorts remain iodine-insufficient. For example, Irish nulliparous women (median urinary iodine concentration [UIC] ≈ 125 µg/L) and a U.S. Mid-Michigan cohort, in which 23% of participants had inadequate intake, illustrate persistent population-level gaps [63,64]. By contrast, Taiwanese women in the first trimester were generally iodine-sufficient (median UIC ≈ 161 µg/L); however, nulliparity and non-use of iodine-containing multivitamins emerged as significant risk factors for deficiency [65].

### 7.5. Supplementation Evidence

Randomized trials in mildly deficient populations (Sweden, India/Thailand) have generally failed to demonstrate cognitive benefits for offspring, reinforcing the notion that timing, dose and baseline status critically influence outcomes [66,67].

In conclusion, adequate iodine status during pregnancy is essential for optimal fetal brain development, while both insufficient and excessive iodine exposure may impair neurodevelopment.

## 8. Public Health Interventions and Iodine Fortification Strategies

### 8.1. Key Public Health Interventions for Iodine Deficiency

Salt iodization, which involves fortifying household and industrial salt with iodine, remains a key public health strategy for the prevention of iodine deficiency and is particularly effective in settings where habitual dietary iodine intake is insufficient [22,68]. Endorsed by the WHO as a cost-effective intervention, its implementation should be adapted to local dietary patterns, environmental iodine availability and population iodine status, to ensure both efficacy and safety [22]. In settings where salt intake is low or actively regulated, iodine fortification of other commonly consumed foods such as flour, drinking water, or condiments, using potassium iodide or iodate, can provide an important complementary strategy [69]. In addition, targeted iodine supplementation, via capsules or iodized oil, may be considered for pregnant women, school-age children, and other high-risk groups when dietary intake is insufficient, as a complement to diet and iodized salt. Some trials have shown that this can improve educational attainment and adult economic outcomes [68].

### 8.2. Health and Economic Impacts

Global clinical IDD prevalence declined from 13.1% to 3.2% following the implementation of salt iodization programs which prevented ~720 million cases and averted 20.5 million newborn cases each year [70].The resulting gains in cognitive development translate into an estimated worldwide economic benefit of approximately $33 billion, largely attributed to higher future earnings [67,69]. A Danish risk–benefit analysis predicts a net health gain of ~35 DALY per 100,000 adults, driven largely by preventing low IQ in children due to maternal iodine deficiency [71].

### 8.3. Implementation Challenges

Successful implementation of iodized salt policies depends on robust supply chains and active private-sector participation, as functional salt production and distribution networks are required to achieve broad population coverage. In low-income settings, weak institutional capacity can limit access among poorer households [68].

Careful monitoring is essential, as iodine intake must remain within a narrow physiological range to avoid both deficiency and excess. Excessive intake may induce hyperthyroidism, underscoring the need for regular surveillance of urinary iodine concentrations and iodine content in salt [22,72].

### 8.4. Overall Strategy and Challenges

Combining salt iodization with selective fortification of other foods and targeted supplementation, backed by robust monitoring systems, offers the most comprehensive approach to preventing iodine deficiency. However, iodine supplementation should be considered only after careful assessment of iodine intake and should be used as a complementary intervention to diet and iodized salt when necessary.

A major challenge of iodine fortification strategies based on salt iodization is their intersection with salt-reduction policies. High dietary salt (sodium chloride) consumed in the kitchen/at the table is a major driver of elevated blood pressure. The WHO estimates that approximately 30% of hypertension cases are caused by excess salt and that a global reduction to <5 g/day would markedly lower hypertension prevalence [55]. However, because iodized salt remains the primary source of iodine in many populations, reduced salt intake may unintentionally lower iodine intake and increase concerns about the long-term adequacy of iodine nutrition. As population salt intake declines, countries must periodically reassess and adjust iodine concentrations in salt to maintain adequate iodine intake and ensure the long-term sustainability of iodine fortification programs, based on monitoring using median urinary iodine concentration at the population level [22].

A WHO document addressing iodine intake in the context of salt-reduction strategies emphasized that efforts to reduce excessive salt consumption and policies promoting salt iodization are both essential and fully compatible [73]. The document further underscored that the safety and effectiveness of iodized salt as a vehicle for iodine intake should not be used to justify maintaining high levels of salt consumption.

Potential interactions between iodine compounds and other micronutrients should be considered in fortification strategies. In particular, iodate used in iodized salt may react with ferrous iron (Fe^2+^) in iron-fortified foods, potentially affecting iodine stability and bioavailability. In addition, iodized salt may contain anti-caking agents such as ferrocyanide salts, which are generally regarded as safe within regulated limits; however, their presence highlights the importance of careful formulation, monitoring, and quality control to ensure the efficacy and safety of combined fortification approaches. Proper storage of iodized salt is important to minimize iodine losses, as iodine may be gradually lost through exposure to moisture, heat, and light; therefore, storage in sealed containers is recommended to preserve iodine content [74].

## 9. Conclusions

Salt iodization has substantially reduced iodine deficiency disorders, yet inadequate monitoring can lead to regions experiencing both deficiency-related hypothyroidism and excess-related thyroid dysfunction. Optimal iodine status—neither deficient nor excessive—is therefore critical for maintaining normal thyroid hormone production and preventing both hypo- and hyperthyroid disease.

Despite salt iodization efforts reducing ID in many regions, mild-to-moderate deficiency remains common, especially in pregnant women and populations with diets insufficient in iodine and limited access to iodized salt, underscoring the need for continued monitoring using urinary iodine biomarkers at the population level and context-specific strategies to ensure adequate iodine intake.

Furthermore, the coexistence of salt reduction policies and salt iodization creates a public health dilemma, as reduced salt intake may compromise iodine nutrition. Integrated strategies are therefore required to maintain adequate iodine intake while achieving cardiovascular health goals.

## Figures and Tables

**Table 1:** Recommended daily iodine intake by age and physiological status.

Population Group	Age Range	Recommended Intake (µg/day)
Infants	0–6 months	110
	7–12 months	130
Children	1–8 years	90
	9–13 years	120
Adolescents and Adults	≥14 years	150
Pregnant Women	-	220–250
Lactating Women	-	250–290

Adapted from [22].

**Table 2:** Harmonized average requirements of iodine by age and physiological status.

Population Group	Age Range	Harmonized Average Requirement (µg/day)
Infants	7–11 months	-
Children	1–10 years	65
Adolescents and Adults	11–14 years	73
	>15 years	95
Pregnancy	Any	160
Lactation	Any	209

Adapted from [24].

## Data Availability

No new data were created or analyzed in this study. Data sharing is not applicable to this article.

## Abbreviations

The following abbreviations are used in this manuscript:

CaCC	Calcium-activated chloride channel.
DIT	Diiodotyrosine.
fT4	Free thyroxine.
HOI	Hypoiodous acid.
I^−^	Iodide.
I_2_	Iodine.
ID	Iodine deficiency.
IDD	Iodine deficiency disorders.
IL	Interleukin.
MIT	Monoiodotyrosine.
NIS	Sodium-iodide symporter.
RNI	Recommended nutrient intakes.
SIC	Salivary iodide concentration.
Tg	Thyroglobulin.
TH	Thyroid hormone.
Th1	T helper 1.
Th2	T helper 2.
TPO	Thyroid peroxidase.
Treg	Regulatory T cell.
TSH	Thyroid-stimulating hormone.
T3	Triiodothyronine.
T4	Thyroxine.
UIC	Urinary iodine concentration.
UIE	Urinary iodine excretion.
WHO	World Health Organization.
